# P53 suppresses the progression of hepatocellular carcinoma via miR‐15a by decreasing OGT expression and EZH2 stabilization

**DOI:** 10.1111/jcmm.16792

**Published:** 2021-09-12

**Authors:** Zhenyu You, Dandan Peng, Yixin Cao, Yuanzhe Zhu, Jianjun Yin, Guangxing Zhang, Xiaodong Peng

**Affiliations:** ^1^ Department of Oncology The First Affiliated Hospital of Nanchang University Nanchang China

**Keywords:** EZH2, hepatocellular carcinoma, miR‐15a, O‐GlcNAc, OGT, P53

## Abstract

Existing literature has highlighted the tumour suppressive capacity of microRNA‐15a (miR‐15a); however, its role in hepatocellular carcinoma (HCC) remains relatively unknown. This study aimed to investigate the role of miR‐15a in HCC and the associated underlying mechanism. Initially, RT‐qPCR was performed to detect the expression of miR‐15a in HCC tissues and cells. Bioinformatics analysis, Pearson correlation coefficient, dual‐luciferase reporter assay, and molecular approaches were all conducted to ascertain the interaction between miR‐15a and O‐linked N‐acetylglucosamine (GlcNAc) transferase (OGT). PUGNAc treatment and cycloheximide (CHX) assay were performed to evaluate O‐GlcNAc and the stabilization of the enhancer of zeste homolog 2 (EZH2). Finally, gain‐ and loss‐of‐function studies were employed to elucidate the role of P53 and the miR‐15a/OGT/EZH2 axis in the progression of HCC, followed by in vivo experiments based on tumour‐bearing nude mice. Our results demonstrated that the miR‐15a expression was decreased in the HCC tissues and cells. P53 upregulated miR‐15a expression, which inhibited the proliferation, migration and invasion of HCC cells, while inducing apoptosis and triggering a G0/G1 cell cycle phase arrest. OGT stabilized EZH2 via catalysing O‐GlcNAc, which reversed the effect of P53 and miR‐15a. The results of our in vivo study provided evidence demonstrating that P53 could suppress the development of HCC via the miR‐15a/OGT/EZH2 axis. P53 was found to inhibit the OGT expression by promoting the expression of miR‐15a, which destabilized EZH2 and suppressed the development of HCC. The key findings of our study highlight a promising novel therapeutic strategy for the treatment of HCC.

## 1 INTRODUCTION

Primary liver cancer is often manifested in the form of hepatocellular carcinoma (HCC) stemming from liver hepatocytes and ranks as the 4^th^ most common malignancy worldwide.^1^, ^2^ HCC remains a leading cause of morbidity and mortality in the western world in regions such as the United States, which has been reported to have the largest increase in cancer‐related deaths over the past 2 decades, resulting in a considerable public health burden.^3^, ^4^, ^5^ In the Asia‐Pacific region, approximately 75% of HCC cases reportedly stem from Asia, highlighting a public health issue.^6^ The risk factors contributing to the occurrence of HCC include viral infections including hepatitis B or C viruses, alcohol assumption‐related cirrhosis, non‐alcoholic steatohepatitis (NASH), non‐alcoholic fatty liver disease (NAFLD), and so forth.^7^ Recently, the next‐generation sequencing (NGS) opens the door to understand the genetic landscape like mutations in liver cancer. Li and Mao^8^ have reported somatic mutations C:G>T:A transitions for example in HCC. Nakagawa and Shibata^9^ have delineated TP53 as a frequent mutation in hepatitis B virus (HBV)–related HCC. HCC treatments are comprised of liver resection, transplantation, radiation, chemotherapy, targeted therapy and immunotherapy.^6^ Over the past decade, although significant improvement regarding prevention, diagnosis and treatment of HCC has occurred with positive outcomes often seen in the early stages of the disease, reoccurrence still occurs in many patients, highlighting the need for an improved understanding of the mechanism underlying the initiation and progression of HCC.^10^

MiRNAs (miRNAs) represent endogenous small non‐coding RNAs that are approximately 22 nucleotides in length and bind to the 3′‐untranslated region (3′‐UTR) of target mRNAs resulting in mRNA degradation or inhibition of mRNA translation.^11^ MiRNAs have been implicated in the progression of a wide variety of diseases including numerous malignancies, Alzheimer's disease and cardiovascular disease^12^, ^13^, ^14^ while the dysregulation of miRNAs been linked with the tumorigenesis of HCC.^15^, ^16^ Liu et al.^17^ reported that microRNA‐15a (miR‐15a), a tumour suppressor, plays a key role in the proliferation, invasion, apoptosis and drug resistance of cancer cells. Recently, miR‐15a has been reported to inhibit migration and invasion of HCC via repressing cMyb, which is implicated as a target for HCC treatment.^18^ Interestingly, miR‐15a has been reported to be one of the downstream tumour suppressors by TP53.^19^ TP53 mutations often occur in hepatitis B virus (HBV)–related HCC.^9^ The dysregulation of miR‐15a‐5p and miR‐26a‐5p promotes the proliferation of ccRCC in regulation of OGT.^20^ OGT represents a GlcNAc transferase, which catalyses serine and threonine residues of intracellular proteins with O‐GlcNAc modification.^21^

In the current study, following a bioinformatics analysis as well as a literature review, we set out to elucidate the role of miR‐15a and the underlying mechanism associated with the regulation of HCC development both in vitro and in vivo to provides a novel therapeutic strategy for the treatment of HCC, through gain‐ and loss‐of‐function analyses, cellular and molecular approaches, and in vivo experiments.

## 2 METHODS

### 2.1 Clinical samples

One hundred and fifty‐three patients following radical hepatectomy at the hepatology department of The First Affiliated Hospital of Nanchang University from 06/2014 to 06/2016 were recruited for the purposes of the current study. All patients were yet to receive any anti‐tumour therapy prior to their operations, including NASH‐related HCC patients (*n* = 26), alcohol‐related liver disease (ALD)–related HCC patients (*n* = 31), hepatitis C virus (HCV)–related HCC patients (*n* = 73) and HBV‐related HCC patients (*n* = 23). All HCC samples isolated from tumour centre were confirmed to be free of necrosis and bleeding while the corresponding adjacent normal tissue samples (no cancer cells confirmed by pathology) were stored at −80℃ for gene expression and histology analysis purposes. Informed consent documentation was signed by all participating patients, with the protocols involving humans performed in strict accordance with the 1964 *Helsinki Declaration*. Patients’ information and follow‐up were collected by medical record room. The outcome of patients after treatment was recorded and added to their clinical records, with the follow‐up process initiating after operative procedures and continued for 36 months. Kaplan–Meier model was used to analyse the correlation between expression of miR‐15a and patients’ overall survival (OS) and disease‐free survival (DFS).

### 2.2 Cell culture and transfection

Hepatocellular carcinoma cell lines HCC‐LM3 and Huh‐7 and normal liver cell line THLE‐2 were purchased from Bluefcell Biotechnology Development Co., Ltd. HCC‐LM3 and Huh‐7 cells were cultured in Dulbecco's modified Eagle medium (DMEM) (Gibco) supplemented with 10% foetal bovine serum (FBS) (Gibco), 100 μg/ml streptomycin and 100 U/ml penicillin. THLE‐2 was cultured in Roswell Park Memorial Institute‐1640 (RPMI‐1640) supplemented with 10% foetal bovine serum (FBS) (Gibco), 100 μg/ml streptomycin and 100 U/ml penicillin. The cells were trypsinized and seeded into 6‐well plates with a density of 1 × 10^5^ cells/well upon reaching the logarithmic phase, followed by culturing. Upon reaching 75% confluency, cells were transiently transfected with Lipofectamine 2000 (Invitrogen) as per the manufacture's protocol. Transfection was performed in groups based on the following: miR‐NC (miRNA control), miR‐15a‐mimic (miR‐15a analog), oe‐NC (overexpression control plasmid), oe‐P53 (overexpression of P53 plasmid), si‐NC (small interfering RNA control), si‐P53 (P53 silencing plasmids, si1‐P53, si2‐P53 and si3‐P53 (synthesized by Shanghai GenePharma Co., Ltd.), oe‐NC + miR‐NC (overexpression plasmid and miRNA control), miR‐15a‐mimic + oe‐NC (miR‐15a‐mimic plasmid and overexpression control plasmid), oe‐OGT + miR‐15a‐mimic (overexpression of OGT plasmid and miR‐15a‐mimic), oe‐OGT (overexpression of OGT plasmid), sh‐OGT (OGT silencing plasmids, sh1‐OGT, sh2‐OGT, and sh3‐OGT (synthesized by Shanghai GenePharma Co., Ltd), si‐EZH2 (OGT silencing plasmids, si1‐EZH2, si2‐EZH2 and si3‐EZH2 (synthesized by Shanghai GenePharma Co., Ltd), oe‐NC + miR‐NC (overexpression control plasmid and miR‐NC), oe‐OGT + si‐EZH2 (overexpression of OGT plasmid and si‐EZH2), oe‐P53 + oe‐NC (overexpression of P53 plasmid and empty control) and oe‐P53 + oe‐EZH2 (overexpression of P53 plasmid and overexpression of EZH2 plasmid). Following a 48‐h period of transfection, real‐time quantitative polymerase chain reaction (RT‐qPCR) and Western blot were performed to analyse the expression of the target genes as well as the corresponding proteins. Plasmids as well as the mimics were synthesized and purchased from Sino Biological Inc. The medium was renewed 6 h after transfection, after which the cells were cultured for an additional 48 h for subsequent experiment purposes.

### 2.3 RNA isolation and RT‐qPCR

Total RNA was extracted using a RNeasy Mini Kit (Qiagen). Reverse transcription (RT) of mRNA was performed using a RT kit (RR047A, Takara) for cDNA collection purposes. miRNA RT was performed using miRNA First Strand cDNA Synthesis (Tailing Reaction) kit (B532451‐0020, Sangon Biotech Co., Ltd.). Samples for RT‐qPCR were prepared with SYBR^®^ Premix Ex Taq™ II (Perfect Real Time) kit (DRR081, Takara) followed by RT‐qPCR (ABI 7500, ABI). The two‐step RT‐qPCR program was performed using pre‐denaturation at 95℃ for 30 s, then 95℃ for 5 s and 60℃ for 34 s with 40 cycles. Each sample was prepared with triplicates. miRNA universe primer and U6 forward primer were provided in miRNA First Strand cDNA Synthesis (Tailing Reaction) kit; other primers were synthesized by Sangon Biotech Co., Ltd. listed in Table S1. Ct value was recorded in each well, glyceraldehyde 3‐phosphate dehydrogenase (GAPDH) or U6 were employed as the internal control, with the 2^−ΔΔCt^ method applied to calculate the relative mRNA expression level. ΔΔCt = (Ct_average of experimental target gene_ − Ct _average of internal control_) − (Ct _average of control target gene_ − Ct _average of internal control_).

### 2.4 Co‐immunoprecipitation (co‐IP) assay

Co‐IP assay was performed using the Pierce co‐IP kit (Thermo scientific 26149), as per the manufacturer's instructions. The total lysate extracted from HCC cells expressing OGT‐FLAG was IP with FLAG Ab or EZH2 Ab, after which Western blotting was performed using the designated antibodies (Abs). The total lysate of HCC cells treated with TMG was IP with IgG or O‐GlcNAc Ab (RL2), followed by Western blotting with EZH2 Ab, and Ig heavy chain (H chain) was used as loading control. The antibodies used included the following: EZH2 (5246, CST); O‐GlcNAc (ab2739, abcam); Flag (SAB4200071, Sigma‐Aldrich); IgG (NI01‐100UG, Millipore).

### 2.5 Western blot

Total proteins were extracted from cultured cells that were used for sodium dodecyl sulphate‐polyacrylamide gel electrophoresis (SDS‐PAGE) and immunoblot. A bicinchoninic acid assay (BCA) kit (20201ES76, Yeasen Biotech) was performed to quantify the protein concentration. The proteins were subsequently separated by SDS‐PAGE, and transferred onto a polyvinylidene fluoride (PVDF) membrane. The membrane was blocked with 5% bovine serum albumin (BSA) for 1 h at room temperature, followed by incubation with primary antibodies including p53 (ab131442, 1: 1000, Abcam), OGT (ab96718, 1: 2000, Abcam), EZH2 (ab186006, 1: 25000, Abcam), O‐GlcNAc (ab2739, 1: 1000, Abcam) and GAPDH (ab8245, 1: 5000, Abcam) at 4℃ overnight with shaking. The next day, PVDF was washed three times using Tris‐buffered saline tween‐20 (TBST) (5 min per wash) and incubated with horseradish peroxide–conjugated secondary antibody, followed by three additional TBST washes (5 min per wash). The PVDF was developed using enhanced chemiluminescence (ECL). ImageJ 1.48u software (National Institute for Health) was employed to quantify the relative expression level of proteins by calculating the ratio of each protein grey value/grey value of GAPDH loading control. Each experiment was performed 3 times.

### 2.6 3‐(4,5‐Dimethylthiazol‐2‐yl)‐2,5‐diphenyltetrazolium bromide (MTT) assay

The cells were seeded into a 96‐well plate at a density of 1 × 10^4^ cells/well and cultured for 0, 1, 2 and 3 days, respectively. MTT was added to each well, followed by an additional 4 h incubation period at 37℃. Next, SDS‐HCl was added to each well and incubated for 30 min. After the medium had been removed, an EL 800 Universal Microplate Reader (BioTek) was used to determine the optical density (OD) at 540 nm.

### 2.7 Cell colony forming unit

The cells were seeded in a 6‐well plate with a density of 600 cells/well and cultured for 2–3 weeks with occasional observation. When colonies were visible, the cell culture procedure was terminated. The colonies were washed using phosphate‐buffered saline (PBS) and fixed with 1 ml methanol for 15 min at room temperature. Next, 1 ml of crystal violet was added to each well followed by incubation for 30 min. The colonies were washed with deionized water and air‐dried at room temperature. The number of colonies was subsequently determined using a scanning plate reader.

### 2.8 Cell scratch assay

Following a 48‐h period of transfection, a sterile pipette tip was used to scratch the centre of the confluent cells. The detached cells and debris were washed away using PBS. The migration of cells was recorded with a microscope 0 or 24 h after scratching, and ImageJ (National Institute for Health) software was used to calculate the migration rate (MR). Each experiment was repeated at least 3 times.

### 2.9 Transwell assay

The upper layer of the Transwell plate was embedded with Matrigel (BD), followed by incubation at 37℃ for 30 min to solidify the Matrigel. The cells were subsequently cultured in a serum‐free medium for 12 h, after which they were collected and resuspended using serum‐free medium to a density of 1 × 10^5^ cells/ml. Medium with 10% FBS was placed in the bottom layer of the Transwell plate. Next, 100 μl cell suspension was added to the bottom layer and incubated at 37℃ for 24 h. The cells with no invasion were wiped away using a cotton swap, while the invasive cells were fixed with 100% methanol and stained with 1% toluidine blue (Sigma). The stained invasive cells were observed under an inverted microscope with 5 fields randomly selected to record the number of invasive cells. Each experiment was repeated 3 times.

### 2.10 Cell apoptosis by flow cytometry

An Annexin V‐FITC apoptosis kit (Invitrogen) was used to analyse cell apoptosis based on the manufacturer's instructions. Briefly, cell culture medium was renewed with serum‐free medium after transfection. Cells were then collected, washed 3 times with PBS (pH 7.4) and resuspended in a staining buffer. Next, 5 μl Annexin‐V‐FITC and 5 μl propidium iodide (PI) were mixed with cells for 10 min followed by incubation at room temperature. The fluorescence‐activated cell sorting (FACS) flow cytometry system (Scalibur‐Becton Dickinson) was employed to analyse apoptosis. Annexin V–positive and PI‐negative stained cells were considered to be apoptotic cells.

### 2.11 Cell cycle analysis by flow cytometry

The cells were collected from a 6‐well plate and washed with PBS 3 times after 48‐h transfection. The cells were subsequently resuspended with 0.5 ml PBS and fixed with 70% ethanol at 4℃ overnight. The next day, cells were washed 3 times with PBS and incubated with 400 μl CCAA (PI, Engreen) solution and 100 μl RNase A (100 μg/ml) at 4℃ for 30 min in dark. Next, cells were resuspended and analysed using a BD flow cytometry machine. 20,000–30,000 cells were recorded, and the data were analysed using ModFit software.

### 2.12 Dual‐luciferase reporter assay

OGT wild‐type (WT) and mutant (MUT) with putative binding sites of miR‐15a were inserted into psiCHECK‐2 plasmid to obtain luciferase reporters (Promega). miR‐15a‐mimic or NC was co‐transfected into the cells according to the manufacture's protocols provided by Lipofectamine 3000 (Invitrogen). After 48‐h transfection, luciferase activity was determined using a dual‐luciferase reporter system (Promega), from which the values of the experiment were normalized to the luciferase activity of Renilla.

### 2.13 Tumour xenograft experiment with nude mice

HCC‐LM3 and Huh‐7 cells that were transfected with oe‐NC, oe‐P53 and oe‐P53 + oe‐EZH2 plasmids were for inoculation purposes. The cells were resuspended in serum‐free DMEM (Gibco) with a density of 1 × 10^6^ cells/200 μl. Thirty‐six Balb/c mice were randomly separated into 6 group (6 mice of each) and housed under the same conditions. The mice were anaesthetized with ether, followed by transplantation with 200 μl cell suspension/mice into the soft region of the back of the right hind leg subcutaneously. Both the mice as well as tumour inoculation findings were recorded every day followed by euthanasia 4 weeks after inoculation. The tumour was isolated, photographed and weighed. The tumour volume was measured every 4 days and calculated using the following formula: Volume = (length × width^2^)/2. RT‐qPCR was performed to measure the expression of miR‐15, and Western blot was used to analyse the expression of P53, OGT and EZH2 proteins. All animal procedures were performed with the approval of the Animal Care and Use Committee of The First Affiliated Hospital of Nanchang University.

### 2.14 Ki67 test

The tumours were fixed in 10% formaldehyde, embedded in paraffin and made into 5‐μm sections. The sections were subsequently incubation with Ki67 (ab15580, 1: 2000, Abcam), followed by incubation with secondary antibody. The Ki67‐positive cells were photographed under a microscope, with the proliferation index (PI) expressed as the number of Ki67‐positive cells.

### 2.15 Terminal deoxynucleotidyl transferase dUTP nick end labelling (TUNEL) assay

A TUNEL Kit (Jiancheng Bioengineering Institute, Nanjing, China) was used to detect the cell apoptosis in accordance with the manufacturer's instructions. The paraffin‐embedded sections were dewaxed in xylene, followed by rehydration in a graded series of ethanol and PBS. The sections were then permeabilized using proteinase K (20 mg/ml in 10 mmol/L Tris–HCl, pH 7.4–8.0) at 37℃ for 15 min and treated with TdT after washing. Finally, the sections were observed and photographed under a light microscope.

### 2.16 Bioinformatics analysis

The GEO database was used to analyse HCC‐related expression data, miRNA data set GSE41077 including 6 cancerous samples and 2 normal samples, mRNA data sets GSE46408 and GSE14520. GSE46408 includes 6 cancerous samples and 6 normal samples, while GSE14520 includes 22 cancerous sample and corresponding normal samples. R package ‘limma’ was used to do differential analysis. For GSE41077, the differential expressed miRNAs were screened based on |logFC| > 1 and *p* < 0.05. For GSE46408, the differentially expressed mRNAs were screened based on logFC| > 0.8 and *p* < 0.05. The expression of target genes in HCC and normal samples as well as the survival curve of patients with HCC was obtained using the ualcan website (http://ualcan.path.uab.edu/index.html). Starbase (http://starbase.sysu.edu.cn/index.php), mirDIP (http://ophid.utoronto.ca/mirDIP/index.jsp#r) and miRDB (http://www.mirdb.org/) were employed to predict the downstream targets of miR‐15a, which intersected with the mRNA differential analysis data.

### 2.17 Statistical analysis

All experimental data were analysed using SPSS 21.0 (IBM Corp.). Quantitative data were expressed as the mean ± standard deviation (mean ± SD). Data from both tumour and adjacent tissues were analysed by paired *t* test. Data between two groups were compared using an unpaired *t* test, while data among multiple groups were analysed via one‐way analysis of variance (anova) and Tukey's post hoc test. Data at different time points were evaluated using repeated measures anova and Bonferroni post hoc test. Kaplan–Meier method was applied to calculate the survival rate, while a log‐rank test was performed for single‐factor analysis purposes. Pearson correlation coefficient was conducted to determine the correlation between samples. Statistically significant difference was indicated by *p* < 0.05.

## 3 RESULTS

### 3.1 miR‐15a expression is downregulated in HCC tissues and cells and correlated with patients’ survival

**FIGURE 1:**
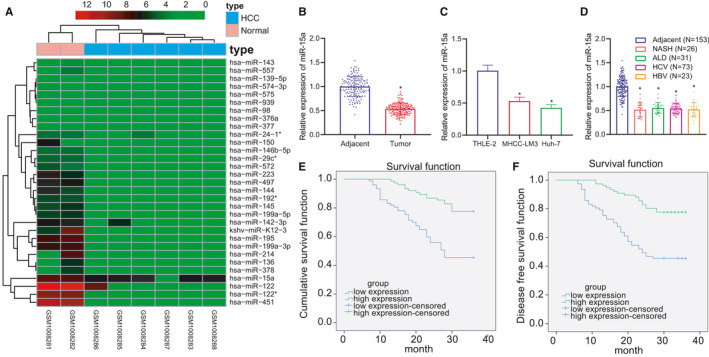
miR‐15a expression was downregulated in HCC tissues and cells and correlated with patients’ survival. (A) Heat map of GSE41077 data set to screen differentially expressed miRNAs in HCC samples. *X*‐axis presents sample numbers, and *y*‐axis presents miRNA. (B) RT‐qPCR to detect the expression of miR‐15a in HCC samples and adjacent healthy tissues, *n* = 153. (C) The expression of miR‐15a in NASH‐related HCC patients (*n* = 26), ALD‐related HCC patients (*n* = 31), HCV‐related HCC patients (*n* = 73) and HBV‐related HCC patients (*n* = 23) determined using RT‐qPCR. (D) RT‐qPCR to detect the expression of miR‐15a in HCC cell lines, HCC‐LM3 and Huh‐7 and normal liver cell line, THLE‐2. (E and F) Kaplan‐Meier method to analyse the correlation between miR‐15a expression and patients’ DFS and OS

The role of miR‐15a has been highlighted in a wide variety of biological processes including cell proliferation, apoptosis, invasion and drug resistance in tumours through binding to the 3′‐UTR of mRNA of target genes.^17^ miR‐15 has been reported to suppress the invasion of liver cancer cells, which makes it a potential therapeutic target of liver cancer.^18^ The miRNA expression data set, GSE41077 was evaluated using |logFC| > 1 and *p* < 0.05 as the miRNA screening criteria R package ‘limma’ was applied to analyse the differentially expressed genes, which revealed 31 upregulated miRNAs (Figure 1A), including miR‐15a which was consistent with the findings of previous literature existing. However, the role of miR‐15a in the development of HCC and the molecular mechanism remain unclear. Thus, we aimed to elucidate the potential mechanism by which miR‐15a contributes to the progression of HCC.

Initially, RT‐qPCR was performed to detect the expression of miR‐15a in 153 cases of HCC tissues and corresponding adjacent healthy tissues, which revealed that the expression of miR‐15a was significantly diminished in the cancer tissues relative to that of the healthy tissues (Figure 1B). RT‐qPCR demonstrated that the expression of miR‐15a was significantly decreased in NASH‐related HCC patients, ALD‐related HCC patients, HCV‐related HCC patients and HBV‐related HCC patients, suggesting that HCC of different aetiologies did not affect the expression of miR‐15a (Figure 1C). We subsequently detected the expression of miR‐15a in the HCC cell lines, HCC‐LM3 and Huh‐7 and normal liver cell line, THLE‐2, which indicated that the results were consistent with the findings of the tissue experiment (Figure 1D). Kaplan–Meier methodology was used to analyse the correlation between miR‐15a expression and patients’ DFS and OS. The results revealed that the OS and DFS in patients with high expression of miR‐15a were notably longer compared with patients with low expression of miR‐15a (Figure 1E,F), indicating that low levels of miR‐15a expression is correlated with poor prognosis in HCC.

### 3.2 miR‐15a overexpression inhibits the proliferation, migration and invasion of live cancer cells

**FIGURE 2:**
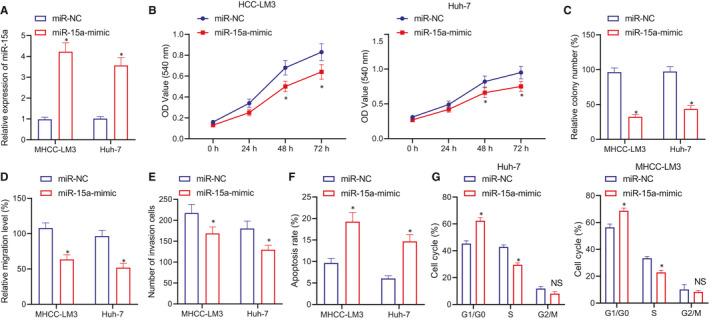
miR‐15a inhibits the proliferation, migration and invasion of live cancer cells. (A) RT‐qPCR to detect the expression of miR‐15a in response to miR‐NC and miR‐15a‐mimic of HCC‐LM3 and Huh‐7 cells. (B) MTT assay to analyse the proliferation of HCC‐LM3 and Huh‐7 cells in miR‐15a in response to miR‐NC and miR‐15a‐mimic. (C) Colony forming unit to analyse the proliferation of HCC‐LM3 and Huh‐7 cells in miR‐15a in response to miR‐NC and miR‐15a‐mimic. (D) Cell scratch assay to study the migration of HCC‐LM3 and Huh‐7 cells in miR‐15a in response to miR‐NC and miR‐15a‐mimic. (E) Transwell assay to study the invasion of HCC‐LM3 and Huh‐7 cells in miR‐15a in response to miR‐NC and miR‐15a‐mimic. (F) Flow cytometry to evaluate the cell apoptosis of HCC‐LM3 and Huh‐7 cells in miR‐15a in response to miR‐NC and miR‐15a‐mimic. (G) Flow cytometry to analyse the cell cycle of HCC‐LM3 and Huh‐7 cells in miR‐15a in response to miR‐NC and miR‐15a‐mimic. *p < 0.05 compared with miR‐NC and presents no significant difference. Quantitative data were presented as mean ± SD. Data of two groups were processed using unpaired t test, and data at different time points were analysed by repeated measures anova and Bonferroni post hoc test. Experiments were repeated 3 times

To further ascertain the effect of miR‐15a on HCC cells, we transfected miR‐15a‐mimic into HCC cell lines, HCC‐LM3 and Huh‐7 and detect the transfection efficiency (Figure 2A). We subsequently analysed the viability of the transfected cells. MTT assay and colony forming unit revealed that compared with miR‐NC, the cell proliferation was considerably decreased in response to miR‐15a‐mimic (Figure 2B,C and Figure S1A). Cell scratch and Transwell assays revealed that miR‐15a overexpression led to a marked reduction in cell migration and invasion (Figure 2D,E and Figure S1B,C). Flow cytometry methods were performed to analyse cell apoptosis, the results of which suggested that the percentage of Annexin V–positive and PI‐negative cells was significantly increased in response to miR‐15a‐mimic relative to miR‐NC, indicative of elevated cell apoptosis (Figure 2F and Figure S1D). Cell cycle analysis by flow cytometry revealed that the miR‐15a‐mimic treatment triggered G0/G1 cell cycle arrest (Figure 2G and Figure S1E). Taken together, the aforementioned results suggest that overexpression of miR‐15a suppresses the proliferation, migration and invasion of live cancer cells, induces cell apoptosis and causes cell cycle G0/G1 arrest.

### 3.3 P53 regulates the expression of miR‐15a to suppress the proliferation, migration and invasion of live cancer cells

**FIGURE 3:**
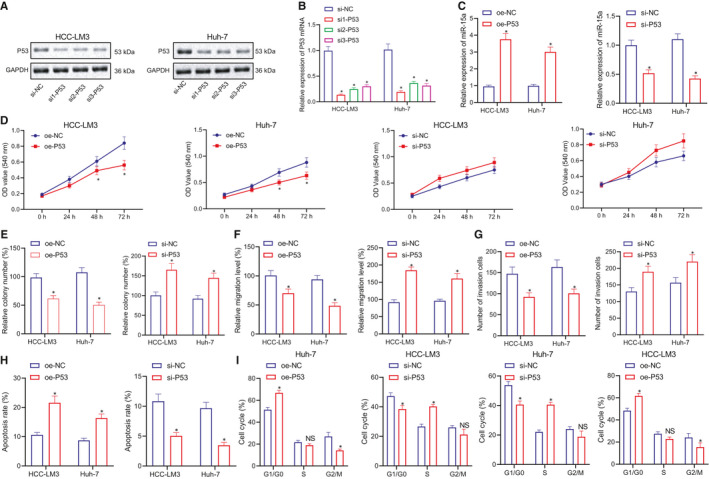
P53 regulates the expression of miR‐15a, which suppresses the proliferation, migration and invasion of live cancer cells. (A and B) Western blot and RT‐qPCR to detect the expression of P53 protein and mRNA in HCC‐LM3 and Huh‐7 cells using si‐NC, si1‐P53, si2‐P53 and si3‐P53. (C) Western blot and RT‐qPCR to detect the expression of miR‐15a mRNA and P53 protein in HCC‐LM3 and Huh‐7 cells in response to oe‐NC and oe‐P53 groups and si‐NC and si‐P53. (D) MTT assay to detect the proliferation of HCC‐LM3 and Huh‐7 cells in miR‐15a in response to oe‐NC and oe‐P53 groups and si‐NC and si‐P53. (E) colony forming unit assay to study the proliferation of HCC‐LM3 and Huh‐7 cells in response to oe‐NC and oe‐P53 groups and si‐NC and si‐P53. (F) Cell scratch assay to study the migration of HCC‐LM3 and Huh‐7 cells of miR‐NC and miR‐15a‐mimics groups. (G) Transwell assay to study the invasion of HCC‐LM3 and Huh‐7 cells in response to oe‐NC and oe‐P53 groups and si‐NC and si‐P53. (H) Flow cytometry to evaluate the cell apoptosis of HCC‐LM3 and Huh‐7 cells in response to oe‐NC and oe‐P53 groups and si‐NC and si‐P53. (I) Flow cytometry to analyse the cell cycle of HCC‐LM3 and Huh‐7 cells in response to oe‐NC and oe‐P53 groups and si‐NC and si‐P53. Quantitative data were presented as mean ± SD. Data of two groups were processed using unpaired *t* test, and data among multiple groups were analysed via one‐way anova and Tukey's post hoc test. Data at different time points were analysed by repeated measures anova and Bonferroni post hoc test. *p < 0.05 compared with oe‐NC and si‐NC, and ns presents no significant difference. Experiments were repeated 3 times

Next, to evaluate the upstream and downstream regulation of miR‐15a, we conducted a literature review, which revealed that the expression of miR‐15a was regulated by P53 in colon and breast cancers.^22^, ^23^ Based on these findings, we set out to verify whether the expression of miR‐15a is regulated by P53 and determine whether it influences the proliferation, migration and invasion of live cancer cells, we overexpressed or silenced P53 in HCC‐LM3 and Huh‐7 cell lines. First, HCC‐LM3 and Huh‐7 cells were transfected with 3 si‐P53 to silence the expression ofP53. RT‐qPCR and Western blot were performed to detect the mRNA and protein expression of P53, which demonstrated that compared with si‐NC, the mRNA and protein levels of P53 by si‐P53 were both downregulated (*p* < 0.05), whereby si1‐P53 exhibited the best silencing efficiency and was selected for subsequent experiments (Figure 3A,B).

The HCC‐LM3 and Huh‐7 cells were subsequently transfected with oe‐P53 and si‐P53. Western blot was performed to detect the expression of P53 protein, after which RT‐qPCR methods were used to detect the expression of miR‐15a. The results indicated that the expression of P53 protein and the mRNA expression of miR‐15a were both markedly elevated in response to oe‐P53 relative to oe‐NC (Figure 3C). Next, we analyse the cell viability via MTT assay and colony forming unit assays (Figure S2A), which demonstrated that cell proliferation was notably decreased following oe‐P53 treatment relative to that of oe‐NC treatment; however, when compared with si‐NC, cell proliferation was significantly increased in response to si‐P53 treatment (Figure 3D,E). Cell scratch and Transwell assays demonstrated that oe‐P53 treatment revealed notably decreased the cell migration and invasion in relative to oe‐NC, while compared with si‐NC group, cell migration and invasion was significantly increased in response to si‐P53 (Figure 3F,G and Figure S2B,C). Cell apoptosis was analysed by flow cytometry which revealed that the percentage of Annexin V–positive and PI‐negative cells was notably increased in response to oe‐P53 in compassion to oe‐NC, indicating the increased cell apoptosis. However, si‐P53 treatment could decrease the apoptosis compared with si‐NC (Figure 3H and Figure S2D). The cell cycle data obtained suggested that oe‐P53 triggered a G0/G1 cell cycle arrest, while in compassion to si‐NC, si‐P53 treatment led to a significant increase in the S phase (Figure 3I and Figure S2E). In summary, our data indicate that miR‐15a is regulated by P53 in HCC cell, while the overexpression of P53 was found to elevate the expression of miR‐15a leading to the inhibition of proliferation, migration and invasion of HCC cells and increased cell apoptosis and G0/G1 phase.

### 3.4 miR‐15a directly targets OGT

**FIGURE 4:**
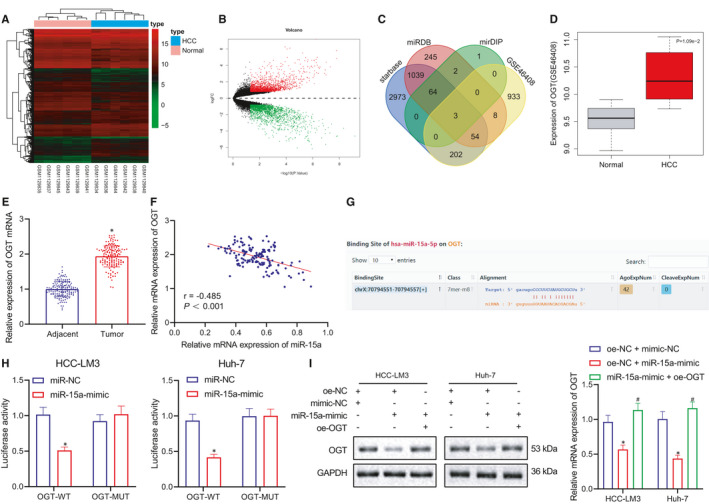
miR‐15a directly targets OGT. (A) Heat map of GSE46408 data set to screen differentially expressed miRNAs in HCC samples. *X*‐axis presents sample numbers, and *y*‐axis presents miRNA. Every square presents the expression of mRNA in a sample. (B) Volcanic map of GSE46408 data set to screen differentially expressed genes in HCC samples. Black colour indicates mRNA without differential expression, green colour indicates downregulated mRNA in HCC samples, and red colour indicates upregulated mRNA in HCC samples. (C) Prediction of miR‐15a downstream targets. Blue dot presents predicted target genes by starbase, red dot presents predicted target genes by miRDB, green dot presents predicted top 70 target genes, yellow dot presents top 1200 significantly upregulated genes predicted by GSE46408, and the central section presents intersection of four databases. (D) The expression of OGT in GSE46408 data set. (E) RT‐qPCR analysis of OGT expression in 153 cases of HCC samples and adjacent healthy tissues. (F) Pearson correlation coefficient analysis of the expression of miR‐15a and OGT mRNA. (G) The binding site between OGT and miR‐15a. (H) Dual‐luciferase reporter assay to analyse the relationship between and miR‐15a and OGT. (I) RT‐qPCR and Western blot to detect the mRNA and protein expression of OGT in response to miR‐NC, miR‐15a mimic, OGT‐WT + miR‐15a mimic HCC cells. Quantitative data were presented as mean ± SD. Data from tumour and the adjacent tissues were analysed by paired *t* test. Data of two groups were processed using unpaired *t* test, and data among multiple groups were analysed via one‐way anova and Tukey's post hoc test. Pearson correlation coefficient was carried out to analyse the correlation between samples. *p < 0.05 compared with adjacent, or miR‐NC, or oe‐NC + mimic‐NC, and ^#^ *p* < 0.05 compared with oe‐NC + miR‐15a‐mimic. Experiments were repeated 3 times

To further investigate the anti‐tumour mechanism of miR‐15a in HCC, we analysed the mRNA expression data set, GSE46408 from the GEO database in addition to |logFC| > 0.8 and *p* < 0.05 employed as the differential miRNA screening criteria. R package ‘limma’ was applied to analyse the differentially expressed genes, which revealed 1754 upregulated miRNA and 1842 downregulated mRNA. Next, the starbase, miRDB and mirDIP databases were explored to predict the downstream targets of miR‐15a, which showed top 70 genes that were intersected with 12,000 upregulated genes from differential mRNA data (Figure 4A–C). The data obtained indicated that CCNE1, MYB and OGT mRNAs existed in these 4 databases. Previous reports have suggested that CCNE1 and MYB are direct targets of miR‐15a^24^, ^25^; however, the relationship between OGT and miR‐15a remains exclusive. Our results indicated that OGT was not only highly expression based on GSE46408 data (Figure 4D) but could also bind with miR‐15a (Figure 4G). Evidence was obtained indicating that the expression of OGT in HCC sample was significantly higher than that of normal samples based on the TCGA database (Figure S3A), and HCC patients with low OGT expression exhibited better prognosis (Figure S3B). OGT upregulation also occurred in HCC samples compared with normal samples according to GSE14520 data set (Figure S3C). In order to verify the aforementioned result, RT‐qPCR was initially performed with 153 patient samples, with the results indicating that OGT was highly expressed in the HCC tissues relative to that of the normal live tissues, which was consistent with data set (Figure 4E). Pearson correlation coefficient highlighted a negative correlation between OGT and miR‐15a (Figure 4F). Next, dual‐luciferase reporter assay was performed to assess the relationship between OGT and miR‐15a, the result of which suggested that miR‐15a‐mimic significantly decreased the luciferase activity of OGT‐WT but not OGT‐MUT (Figure 4H). Finally, Western blot and RT‐qPCR results revealed that miR‐15a‐mimic significantly downregulated the expression of the OGT protein (Figure 4I). Altogether, these results suggest that OGT is a direct target of miR‐15a.

### 3.5 OGT‐mediated O‐GlcNAc stabilizes EZH2 and increases its expression

**FIGURE 5:**
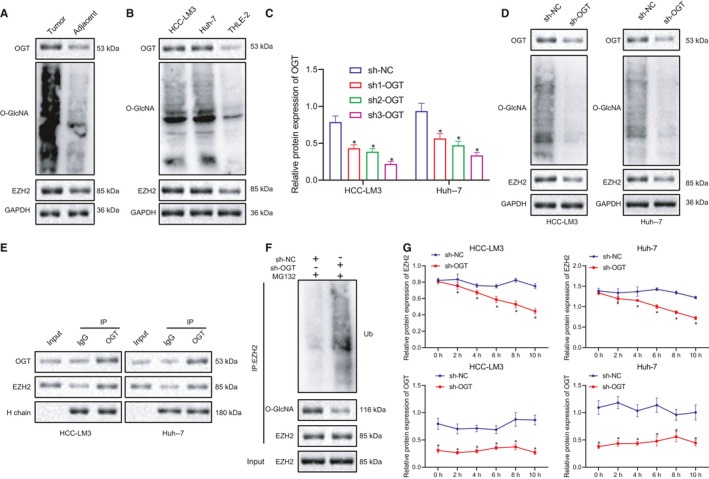
OGT‐mediated O‐GlcNAc stabilizes EZH2 and increases its expression. (A) Western blot to detect the expression of OGT, O‐GlcNAc and EZH2 in HCC tissues and adjacent healthy tissues. (B) Western blot to analyse the expression of OGT, O‐GlcNAc and EZH2 in HCC cell lines, HCC‐LM3 and Huh‐7 and normal liver cell line, THLE‐2. (C) Western blot and RT‐qPCR to detect the expression of OGT in HCC‐LM3 and Huh‐7 cells of sh‐NC, sh1‐OGT, sh2‐OGT and sh3‐OGT. (D) The expression of OGT, O‐GlcNAc, and EZH2 in HCC‐LM3 and Huh‐7 cells after silencing OGT. (E) The total lysate extracted from HCC cells was immunoprecipitated with OGT Ab, followed by Western blot analysis with designated antibodies with Ig heavy chain (H chain) as loading control. (F) Western blot analysis of ubiquitin and O‐GlcNAc on EZH2 immunoprecipitated with HCC cells stably expressing sh‐NC or sh‐OGT in the presence or MG132(10 μM) for proteasome inhibition. (G) Detection of EZH2 and OGT protein levels treated with CHX (50 μg/ml) at different time points. Quantitative data were presented as mean ± SD. Data of two groups were processed using unpaired *t* test, and data among multiple groups were analysed via one‐way anova and Tukey's post hoc test. *p < 0.05 compared with sh‐NC. Experiments were repeated 3 times

Next, to further ascertain the role of OGT in HCC, we conducted a literature search which revealed that OGT could catalyse O‐GlcNAcylation (O‐GlcNAc) on EZH2 and regulate its expression and stability.^26^ To verify if this was relevant to HCC, we initially examined the expression of OGT, EZH2 and O‐GlcNAc in HCC tissues and corresponding adjacent healthy tissues. The results showed that the expression levels of OGT, EZH2 and O‐GlcNAc in HCC tissues were significantly higher than that of in the adjacent healthy tissues (Figure 5A). We subsequently evaluated the expression of OGT, EZH2 and O‐GlcNAc in HCC‐LM3 and Huh‐7 cells and normal liver cell line THLE‐2, which demonstrated that the results were consistent with the results seen in the tissues (Figure 5B).

Next, we transfected 3 sh‐OGT in HCC‐LM3 and Huh‐7 cells to silence its expression. RT‐qPCR and Western blot results uncovered that silencing of OGT markedly reduced the mRNA and protein expression of OGT in comparison to sh‐NC, in which sh3‐OGT showed the lowest level of OGT. Thus, it was selected for the subsequent experiments (Figure 5C). The expression levels of O‐GlcNAc and EZH2 were also significantly decreased in OGT silenced HCC‐LM3 and Huh‐7 cells (Figure 5D). Next, to verify whether EZH2 was modified by glycosylation and whether glycosylation influences EZH2 in HCC cell lines, co‐IP assay was performed and the results indicated that OGT could interact with EZH2 in HCC‐LM3 and Huh‐7 cells (Figure 5E). Moreover, knockdown of OGT reduced the O‐GlcNAc glycosylation level of EZH2 and increased the ubiquitination level (Figure 5F). Meanwhile, CHX analysis demonstrated that silencing OGT could decrease the stability of EZH2 to promote its degradation (Figure 5G), suggesting that OGT stabilizes EZH2 through O‐GlcNAc in HCC cells.

### 3.6 OGT promotes proliferation, migration and invasion of HCC cells by regulating EZH2

**FIGURE 6:**
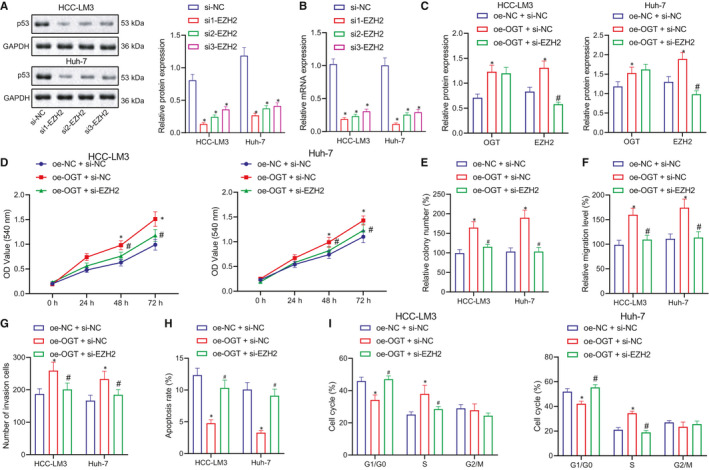
OGT promotes the proliferation, migration and invasion of HCC cells by regulating EZH2. (A and B) Western blot and RT‐qPCR to detect the expression of EZH2 protein and mRNA in HCC‐LM3 and Huh‐7 cells of si‐NC, si1‐EZH2, si2‐EZH2 and si3‐EZH2 groups. (C) Western blot to detect the expression of EZH2 protein and mRNA in HCC‐LM3 and Huh‐7 cells of response to oe‐NC + si‐NC, oe‐OGT + si‐NC and oe‐OGT + si‐EZH2. (D) MTT assay to detect the proliferation of HCC‐LM3 and Huh‐7 cells in response to oe‐NC + si‐NC, oe‐OGT + si‐NC and oe‐OGT + si‐EZH2. (E) Colony forming unit assay to analyse the proliferation of HCC‐LM3 and Huh‐7 cells in response to oe‐NC + si‐NC, oe‐OGT + si‐NC and oe‐OGT + si‐EZH2. (F) Cell scratch assay to study the migration of HCC‐LM3 and Huh‐7 cells in response to oe‐NC + si‐NC, oe‐OGT + si‐NC and oe‐OGT + si‐EZH2. (G) Transwell assay to study the invasion of HCC‐LM3 and Huh‐7 cells in response to oe‐NC + si‐NC, oe‐OGT + si‐NC and oe‐OGT + si‐EZH2. (H) Flow cytometry to evaluate the cell apoptosis of HCC‐LM3 and Huh‐7 cells in response to oe‐NC + si‐NC, oe‐OGT + si‐NC and oe‐OGT + si‐EZH2. (I) Flow cytometry to analyse the cell cycle of HCC‐LM3 and Huh‐7 cells in response to oe‐NC + si‐NC, oe‐OGT + si‐NC and oe‐OGT + si‐EZH2. Quantitative data were presented as mean ± SD. Data among multiple groups were analysed via one‐way anova and Tukey's post hoc test. *p < 0.05 compared with si‐NC or oe‐NC + si‐NC, ^#^ *p* < 0.05 compared with oe‐OGT + si‐NC. Experiments were repeated 3 times

Next, to evaluate whether OGT regulates the expression of EZH2 to influence live cancer progression, OGT was overexpressed or silenced EZH2 in HCC‐LM3 and Huh‐7 cells as follows, oe‐NC + si‐NC, oe‐OGT + si‐NC, and oe‐OGT + si‐EZH2. First, we transfected 3 si‐EZH2 to silence EZH2 expression. RT‐qPCR and Western blot were performed to detect the mRNA and protein expression of EZH2, which suggested that the mRNA and protein expression of EZH2 was markedly downregulated following the silencing of EZH2 silencing compared with si‐NC, in which si1‐EZH2 resulted in the lowest level of EZH2, which was selected for the subsequent experiments (Figure 6A,B). Western blot analysis also demonstrated that the expression of OGT was significantly increased following OGT overexpression, while the expression of EZH2 was considerably decreased after treatment with si‐EZH2 (Figure 6C).

We subsequently applied MTT and colony forming unit assays to study the cell proliferation, cell scratch assay was employed to detect the cell migration, Transwell assay was conducted to detect the cell invasion, and flow cytometry (Figure S4A–E) was performed to analyse the cell apoptosis and cell cycle. The data revealed that cell proliferation, migration and invasion were remarkably increased (Figure 6D–G) and cell apoptosis was markedly decreased (Figure 6H), while the S phase of the cell cycle was considerably increased (Figure 6I) after oe‐OGT treatment alone. Cell proliferation, migration and invasion were all significantly decreased (Figure 6D–G), cell apoptosis was significantly increased (Figure 6H), and cell cycle arrested at the G0/G1 phase (Figure 6I) by the further treatment of si‐EZH2. The aforementioned results suggest that OGT upregulates the expression of EZH2 to promote the progression of HCC cell, while si‐EZH2 inhibits the promotion of liver cell growth by OGT overexpression. Thus, in HCC cell, OGT could regulate EZH2 expression to promote the proliferation, migration and invasion of HCC cells and decrease the apoptosis.

### 3.7 P53 inhibits the proliferation, migration and invasion and increases the apoptosis of HCC cells through miR‐15a/OGT/EZH2 axis

**FIGURE 7:**
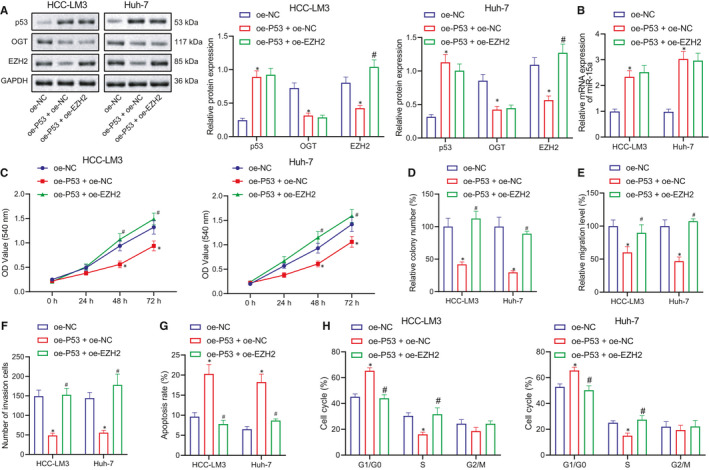
P53 inhibits the proliferation, migration and invasion and increases the apoptosis of HCC cells through miR‐15a/OGT/EZH2 axis. (A) Western blot to detect the expression of P53, OGT and EZH2 proteins in HCC‐LM3 and Huh‐7 cells in response to oe‐NC, oe‐P53 + oe‐NC and oe‐P53 + oe‐EZH2. (B) RT‐qPCR to detect the expression of miR‐15a in HCC‐LM3 and Huh‐7 cells in response to oe‐NC, oe‐P53 + oe‐NC and oe‐P53 + oe‐EZH2. (C) MTT assay to detect the proliferation in HCC‐LM3 and Huh‐7 cells in response to oe‐NC, oe‐P53 + oe‐NC and oe‐P53 + oe‐EZH2. (D) Colony forming unit assay to detect the proliferation in HCC‐LM3 and Huh‐7 cells in response to oe‐NC, oe‐P53 + oe‐NC and oe‐P53 + oe‐EZH2. (E) Cell scratch assay to study the migration in HCC‐LM3 and Huh‐7 cells in response to oe‐NC, oe‐P53 + oe‐NC and oe‐P53 + oe‐EZH2. (F) Transwell assay to study the invasion in HCC‐LM3 and Huh‐7 cell in response to oe‐NC, oe‐P53 + oe‐NC and oe‐P53 + oe‐EZH2. (G) Flow cytometry to evaluate the cell apoptosis in HCC‐LM3 and Huh‐7 cells in response to oe‐NC, oe‐P53 + oe‐NC and oe‐P53 + oe‐EZH2. (H) Flow cytometry to analyse the cell cycle in HCC‐LM3 and Huh‐7 in response to oe‐NC, oe‐P53 + oe‐NC and oe‐P53 + oe‐EZH2. Quantitative data were presented as mean ± SD. Data among multiple groups were analysed via one‐way anova and Tukey's post hoc test. *p < 0.05 compared with oe‐NC, ^#^ *p* < 0.05 compared with oe‐P53 + oe‐NC, and ns indicates no significant difference. Experiments were repeated 3 times

Based on the aforementioned findings, we asserted that P53 regulates miR‐15a/OGT to affect EZH2 expression and finally leads to the suppression of HCC cells. In order to evaluate our hypothesis, we transfected oe‐EZH2 in P53 overexpressing HCC‐LM3 and Huh‐7 cells to ascertain whether EZH2 could reverse the anti‐tumour effect of P53 overexpression. HCC‐LM3 and Huh‐7 cells were grouped as follows, oe‐NC, oe‐P53 + oe‐NC and oe‐P53 + oe‐EZH2. Western blot was performed to detect the expression of P53, OGT and EZH2, while RT‐qPCR was conducted to detect the expression of miR‐15a. The Western blot results demonstrated that the expression of OGT and EZH2 was significantly downregulated in response to oe‐P53 relative to oe‐NC, while only OGT expression was notably decreased after treatment of oe‐EZH2 (Figure 7A). The RT‐qPCR data revealed that relative to oe‐NC, miR‐15a expression was considerably increased following treatment with oe‐P53 or oe‐EZH2 alone or in combination (Figure 7B).

MTT and colony forming unit assays revealed that treatment with oe‐P53 alone led to a notable reduction in the proliferation of HCC cells, while the cell proliferation was recovered by further treatment of oe‐EZH2 (Figure 7C,D). Cell scratch and Transwell assays demonstrated that the migration and invasion of HCC cells were significantly decreased in response to treatment with oe‐P53 alone (Figure 7E,F). Cell apoptosis and cell cycle analyses uncovered that compared with oe‐NC, cell apoptosis was increased (Figure 7G) while the cell cycle was arrested at the G0/G1 phase in response to oe‐P53 alone (Figure 7H). However, oe‐P53 in combination with oe‐EZH2 brought about a decrease in cell apoptosis (Figure 7G) while increasing the S phase of the cell cycle (Figure 7H). These results indicate that P53 suppresses OGT expression by promoting the expression of miR‐15a, inhibiting the stability of EZH2 to inhibit proliferation, migration and invasion while elevating apoptosis and G0/G1 phase arrest in HCC.

### 3.8 P53 suppresses the growth of xenograft liver tumour in nude mice through regulating miR‐15a/OGT/EZH2 axis

**FIGURE 8:**
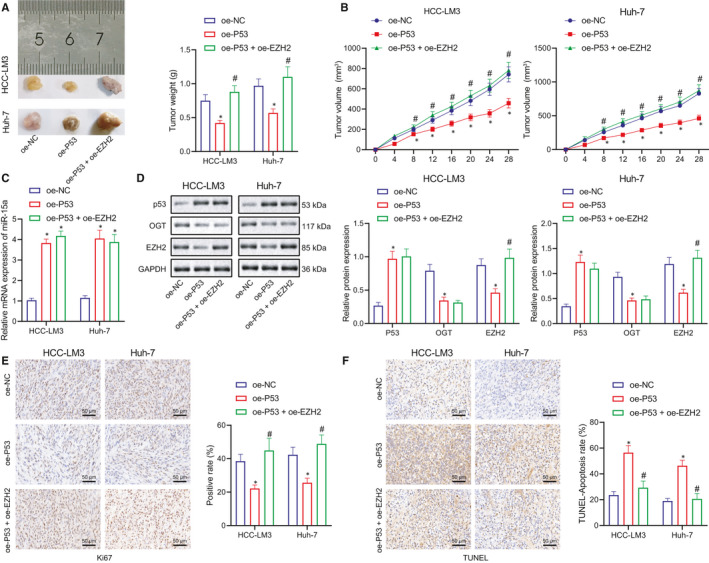
P53 suppresses the growth of xenograft liver tumour in nude mice through regulating miR‐15a/OGT/EZH2 axis. (A) Tumour weights after 4 week's inoculation of HCC cells in nude mice, *n* = 6. (B) The trend of tumour sizes formed by HCC cells within 4 weeks in oe‐P53 + oe‐NC and oe‐P53 + oe‐EZH2 groups, *n* = 6. C, RT‐qPCR to detect the expression of miR‐15a in tumours of nude mice in response to oe‐NC, oe‐P53 + oe‐NC and oe‐P53 + oe‐EZH2, *n* = 6. (D) Western blot to detect the expression of P53, OGT and EZH2 in tumours of nude mice in response to oe‐NC, oe‐P53 + oe‐NC and oe‐P53 + oe‐EZH2. (E) Ki67 to analyse the proliferation of tumour cells. (F) TUNEL to analyse the apoptosis of tumour cells. *p < 0.05 compared with oe‐NC, ^#^ *p* < 0.05 compared with oe‐P53 + oe‐NC. Experiments were repeated 3 times

Next, to further validate whether P53 influences the initiation and development of HCC through miR‐15a/OGT/EZH2 in vivo, we performed tumour‐bearing experiment in nude mice. The mice were inoculated with HCC‐LM3 and Huh‐7 cells transfected with oe‐NC, oe‐P53 and oe‐P53 + oe‐EZH2 plasmids, respectively. The growth of tumours in each group was recorded with the tumour volumes calculated every 4 days. After 28 days, the mice were killed after which their tumours were dissected, weighed and volumes calculated. RT‐qPCR was performed to detect the expression of miR‐15a, and Western blot was used to detect the expression of P53, OGT and EZH2 in tumours. Ki67 was used as a proliferation marker to analyse the proliferation of tumour cells via immunohistochemistry. TUNEL assay was applied to evaluate the apoptosis of the tumour cells. The results demonstrated that relative to oe‐NC, overexpression of P53 significantly decreased the tumorigenic ability in the nude mice, with the tumour volume also notably smaller in the modelled mice (Figure 8A,B), and miR‐15a expression was also considerably elevated (Figure 8C), while the expression levels of OGT and EZH2 were markedly decreased (Figure 8D). Further treatment with oe‐EZH2 increased the tumour volume in mice (Figure 8A,B) and led to a notable upregulation in the expression of EZH2 (Figure 8D). Ki67 immunohistochemistry results revealed that in comparison to oe‐NC, cell proliferation was significantly decreased in response to oe‐P53, while the cell proliferation was considerably increased by the further treatment of oe‐EZH2 (Figure 8E). TUNEL assay revealed that compared with oe‐NC, cell apoptosis was significantly increased in response to oe‐P53, while apoptosis was notably decreased following further treatment with oe‐EZH2 (Figure 8F). Taken together, these results suggest that P53 promotes the expression of miR‐15a to inhibit OGT expression, which inhibits EZH2 stability to inhibit liver tumour growth in nude mice.

## 4 DISCUSSION

HCC remains a notable contributor to cancer‐related deaths on a global scale.^27^ Thus, a deeper understanding of the molecular pathogenesis underpinning HCC is crucial to improving diagnosis, prevention and treatment approaches for the disease. Our data demonstrated that the expression of miR‐15a was downregulated in both HCC tumour tissues and cell lines, while a relationship between miR‐15a and patient prognosis was indicating that miR‐15a might be related to the development of HCC. Gain‐of‐function studies demonstrated that miR‐15a inhibited the proliferation, migration and invasion of HCC cancerous cells, acting to induce apoptosis and trigger a G0/G1 phase arrest. Consistent with the findings of our literature review, bioinformatics analysis, gain‐ and loss‐of‐function study, and molecular approaches, P53 was revealed to be the upstream regulator of miR‐15a, promoting its expression and exerting anti‐HCC effect and GlcNAc OGT as the downstream target of miR‐15a. Further investigation into the role of OGT in HCC revealed that OGT‐mediated O‐GlcNAc stabilized EZH2 to induce its expression, with OGT found to promote the proliferation, migration and invasion of HCC cells, blocked apoptosis and increased the S phase. In contrast, P53 exhibited an opposite effect compared to OGT, which inhibits HCC cell progression through miR‐15a/OGT/EZH2 axis. A HCC mouse xenograft model was established to elucidate the anti‐tumour property of P53 in vivo, with the results indicating that P53 could suppress the growth of xenograft liver tumour in nude mice by regulating miR‐15a/OGT/EZH2 axis.

miRNAs as gene expression modulators have been comprehensively investigated with studies suggesting their participation in the normal and pathologic processes in cells.^28^, ^29^ Accumulating studies continue to emphasize the involvement of miRNAs in the repression of cancer development.^30^, ^31^, ^32^, ^33^ In liver cancer, miR‐15a has been reported to inhibit the cancer cell proliferation and metastasis, which supports our results showing miR‐15a as a tumour suppressor in liver cancer.^18^, ^34^ However, the upstream regulators and downstream targets of miR‐15a that are involved in the progression of HCC are yet to be elucidated clear. In colon and breast cancers, the expression of miR‐15a has been reported to be regulated by the tumour suppressor gene, P53.^22^, ^23^ In HCC, our results demonstrate that the miR‐15a expression level is positively correlated with P53, which in turn suppresses the live cancer development. Consistent with the observations of the current study, LINC00662 was previously reported to promote HCC cell proliferation and cell cycle by competitively binding with miR‐15a, miR‐16 and miR‐107.^35^

Bioinformatics analysis, Pearson correlation coefficient and dual‐luciferase reporter assay results revealed that OGT was a direct target of miR‐15a. P53 is a tumour suppressor that is activated by cellular stress events such as DNA damage and oncogenic stress, which induces cell cycle arrest and apoptosis or senescence.^36^, ^37^ Meanwhile, the tumour‐inhibiting effect of P53 remained unchanged in the mutant status of HCC‐LM3 and Huh‐7.^38^, ^39^ P53, the ubiquitination and degradation of which may be involved in the PRIM1‐promoted proliferation, migration/invasion and sorafenib resistance of HCC cells, has also been reported to induce cancerous cell apoptosis in HCC.^40^, ^41^, ^42^ P53 and miR‐15a share a positive loop, which is important for tumour suppression in HCC by miR‐15a. OGT is a GlcNAc transferase, which catalyses serine and threonine residues of intracellular proteins with O‐GlcNAc modification.^21^ Xu et al.^43^ highlighted the oncogenic role of OGT in fatty liver–associated liver cancer. Our data demonstrated that miR‐15a directly targets OGT, which indicates the oncogenic aspect of OGT in HCC and inspires us to study the mechanism of how OGT contributes to the development of HCC.

EZH2 is the catalytic component of the polycomb repressive complex 2 (PRC2), which catalyses the dimethylation and trimethylation of H3K27 (H3K27me2/3) as well as acting to transcriptionally repress gene expression.^44^ Sasaki et al.^45^ asserted that EZH2 was overexpressed and associated with the malignant progression of HCC. Chu et al.^26^ demonstrated that OGT stabilized EZH2 via catalysing O‐GlcNAc modification, which regulated its function epigenetically. The aforementioned results drove us to investigate whether OGT exerts an oncogenic influence through O‐GlcNAc modification on EZH2. Our data revealed that the expression levels of OGT, O‐GlcNAc and EZH2 were notably upregulated in both HCC tissues and cell lines, while suggesting that O‐GlcNAc stabilizes EZH2 and increases its expression, thereby enhancing HCC progression while inhibiting apoptosis. As illustrated by our results P53 as upstream regulator of miR‐15a may contribute to the anti‐tumour effect of miR‐15a/OGT/EZH2 axis regulated by P53. Gain‐of‐function analysis along with cellular and molecular approaches revealed that P53 suppresses the proliferation, migration and invasion of HCC cells as well as elevating apoptosis via the miR‐15a/OGT/EZH2 axis. Finally, to expand our research *in vivo*, liver tumour‐bearing nude mice models were established. The animal experiments provided evidence verifying that P53 could inhibit liver tumour growth in nude mice by regulating the miR‐15a/OGT/EZH2 axis.

**FIGURE 9:**
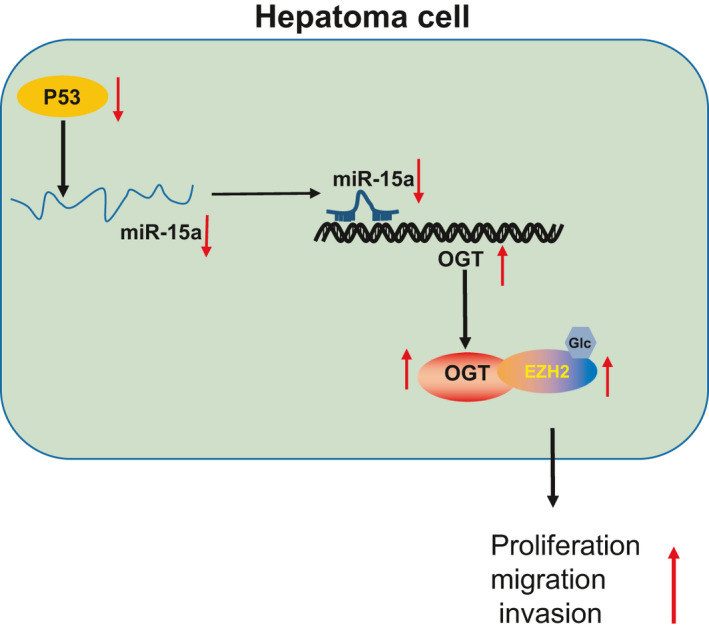
P53 inhibits OGT expression by promoting the expression of miR‐15a, which destabilizes EZH2 and inhibits the proliferation, migration and invasion and increases the apoptosis of HCC cells

In conclusion, the key findings of our study elucidate a novel molecular mechanism whereby miR‐15a functions as an anti‐tumour miRNA in HCC, highlighting a promising therapeutic strategy for the treatment of HCC (Figure 9). Future study is required to demonstrate the mechanism by which P53 regulates the expression of miR‐15a and to ascertain whether miR‐15a exerts anti‐tumour effects on other types of cancers.

## CONFLICTS OF INTEREST

The authors declare no conflict of interest.

## AUTHOR CONTRIBUTIONS

**Zhenyu You:** Conceptualization (equal); Data curation (equal); Writing‐original draft (equal); Writing‐review & editing (equal). **Dandan Peng:** Conceptualization (equal); Data curation (equal); Writing‐review & editing (equal). **Yixin Cao:** Supervision (equal); Writing‐original draft (equal). **Yuanzhe Zhu:** Supervision (equal); Writing‐original draft (equal). **Jianjun Yin:** Resources (equal); Writing‐original draft (equal). **Guangxing Zhang:** Resources (equal); Writing‐original draft (equal). **Xiaodong Peng:** Conceptualization (equal); Data curation (equal); Writing‐review & editing (equal).

## Supporting information

Fig S1

Fig S2

Fig S3

Fig S4

Table S1

## Data Availability

Research data are not shared.
