# Corrigendum

**DOI:** 10.1111/jcmm.17335

**Published:** 2022-06-06

In Xiaodong Peng et al.,^1^ the Figure S2C Transwell and WB experiment of Figure 3A, Figure 4I, Figure 5E, Figure 6A, Figure 7A and Figure 8D are incorrect. The correct figures are shown below. The authors confirm that all results and conclusions of this article remain unchanged.

**FIGURE 3:**
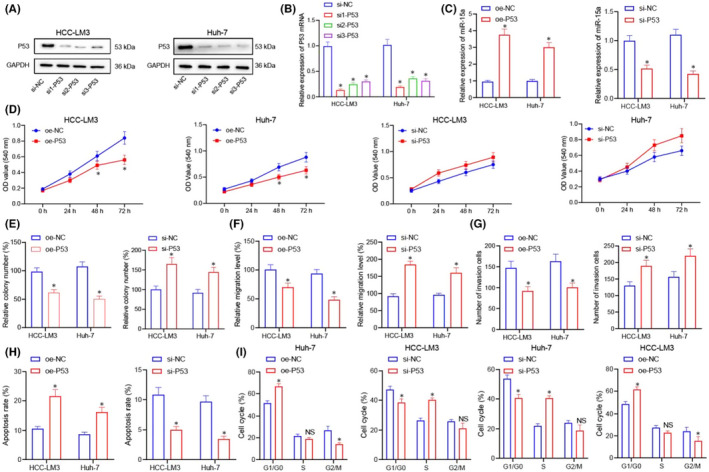
P53 regulates the expression of miR‐15a, which suppresses the proliferation, migration and invasion of live cancer cells. (A and B) Western blot and RT‐qPCR for detecting the expression of P53 protein and mRNA in HCC‐LM3 and Huh‐7 cells using si‐NC, si1‐P53, si2‐P53 and si3‐P53. (C) Western blot and RT‐qPCR for detecting the expression of miR‐15a mRNA and P53 protein in HCC‐LM3 and Huh‐7 cells in response to oe‐NC and oe‐P53 roups and si‐NC and si‐P53. (D) MTT assay for detecting the proliferation of HCC‐LM3 and Huh‐7 cells in miR‐15a in response to oe‐NC and oe‐P53 roups and si‐NC and si‐P53. (E) colony forming unit assay for studying the proliferation of HCC‐LM3 and Huh‐7 cells in response to oe‐NC and oe‐P53 groups and si‐NC and si‐P53. (F) Cell scratch assay for studying the migration of HCC‐LM3 and Huh‐7 cells of miR‐NC and miR‐15a‐mimics groups. (G) Transwell assay for studying the invasion of HCC‐LM3 and Huh‐7 cells in response to oe‐NC and oe‐P53 groups and si‐NC and si‐P53. (H) Flow cytometry for evaluating the cell apoptosis of HCC‐LM3 and Huh‐7 cells in response to oe‐NC and oe‐P53 groups and si‐NC and si‐P53. (I) Flow cytometry for analysing the cell cycle of HCC‐LM3 and Huh‐7 cells in response to oe‐NC and oe‐P53 groups and si‐NC and si‐P53. Quantitative data were presented as mean ± SD. Data of two groups were processed using unpaired t test, and data among multiple groups were analysed via one‐way ANOVA and Tukey's post hoc test. Data at different time points were analysed by repeated measures ANOVA and the Bonferroni post hoc test. *p < 0.05 compared with oe‐NC and si‐NC, and ns presents no significant difference. Experiments were repeated 3 times

**FIGURE 4:**
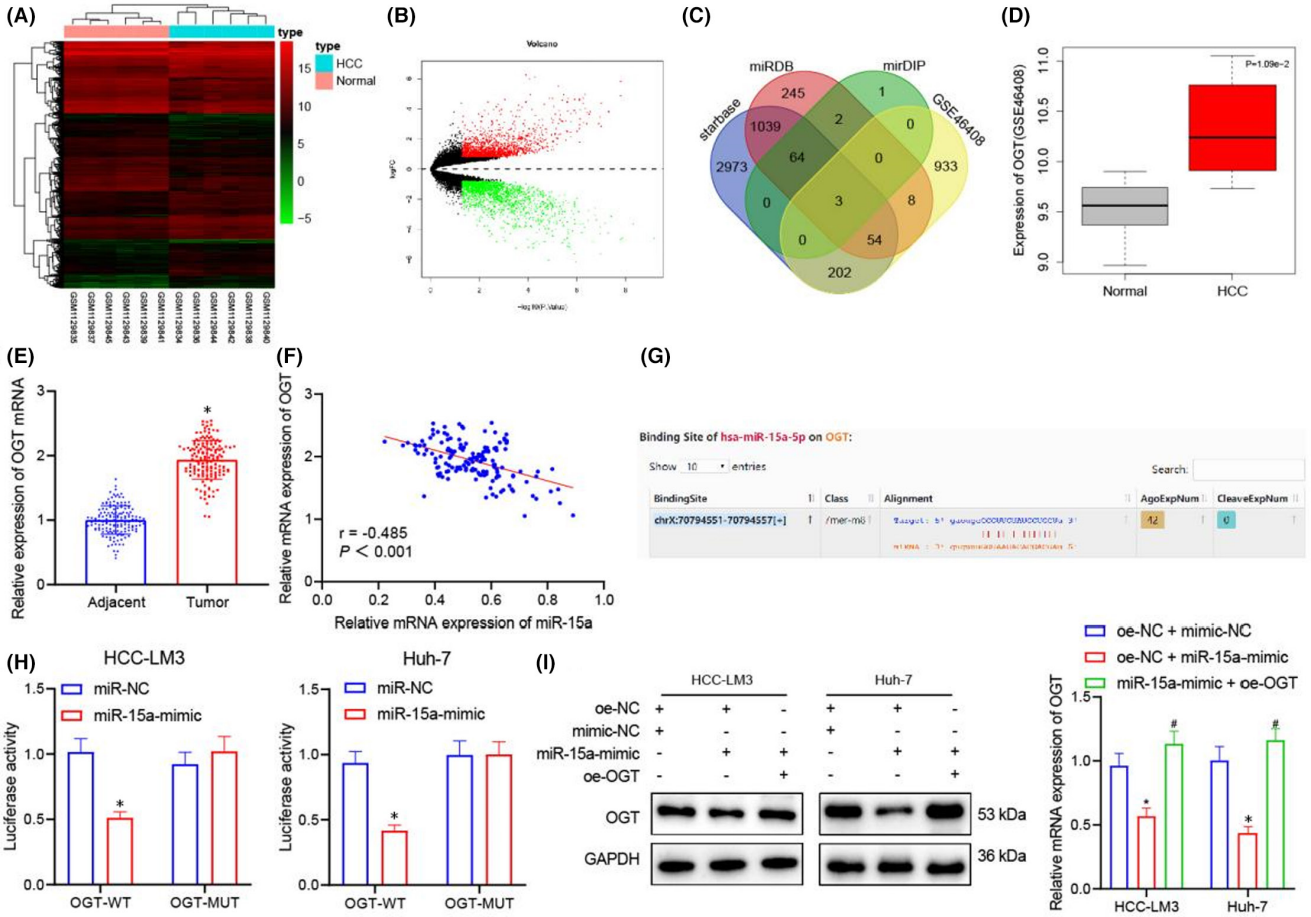
miR‐15a directly targets OGT. (A) Heat map of GSE46408 data set to screen differentially expressed miRNAs in HCC samples. X‐axis represents sample numbers, and y‐axis represents miRNA. Every square represents the expression of mRNA in a sample. (B) Volcanic map of GSE46408 data set for screening differentially expressed genes in HCC samples. Black colour indicates mRNA without differential expression, green colour indicates downregulated mRNA in HCC samples, and red colour indicates upregulated mRNA in HCC samples. (C) Prediction of miR‐15a downstream targets. Blue dot represents predicted target genes by starbase, red dot represents predicted target genes by miRDB, green dot represents predicted top 70 target genes, yellow dot represents top 1200 significantly upregulated genes predicted by GSE46408, and the central section represents intersection of four databases. (D) The expression of OGT in GSE46408 data set. (E) RT‐qPCR analysis of OGT expression in 153 cases of HCC samples and adjacent healthy tissues. (F) Pearson's correlation coefficient analysis of the expression of miR‐15a and OGT mRNA. (G) The binding site between OGT and miR‐15a. (H) Dual‐luciferase reporter assay for analysing the relationship between and miR‐15a and OGT. (I) RT‐qPCR and Western blot for detecting the mRNA and protein expression of OGT in response to miR‐NC, miR‐15a mimic and OGT‐WT +miR‐15a mimic HCC cells. Quantitative data were presented as mean ± SD. Data from tumour and the adjacent tissues were analysed by paired *t* test. Data of two groups were processed using unpaired t test, and data among multiple groups were analysed via one‐way ANOVA and Tukey's post hoc test. Pearson's correlation coefficient was carried out to analyse the correlation between samples. *p < 0.05 compared with adjacent, or miR‐NC, or oe‐NC +mimic‐NC, and # *p* < 0.05 compared with oe‐NC +miR‐15a‐mimic. Experiments were repeated 3 times

**FIGURE 5:**
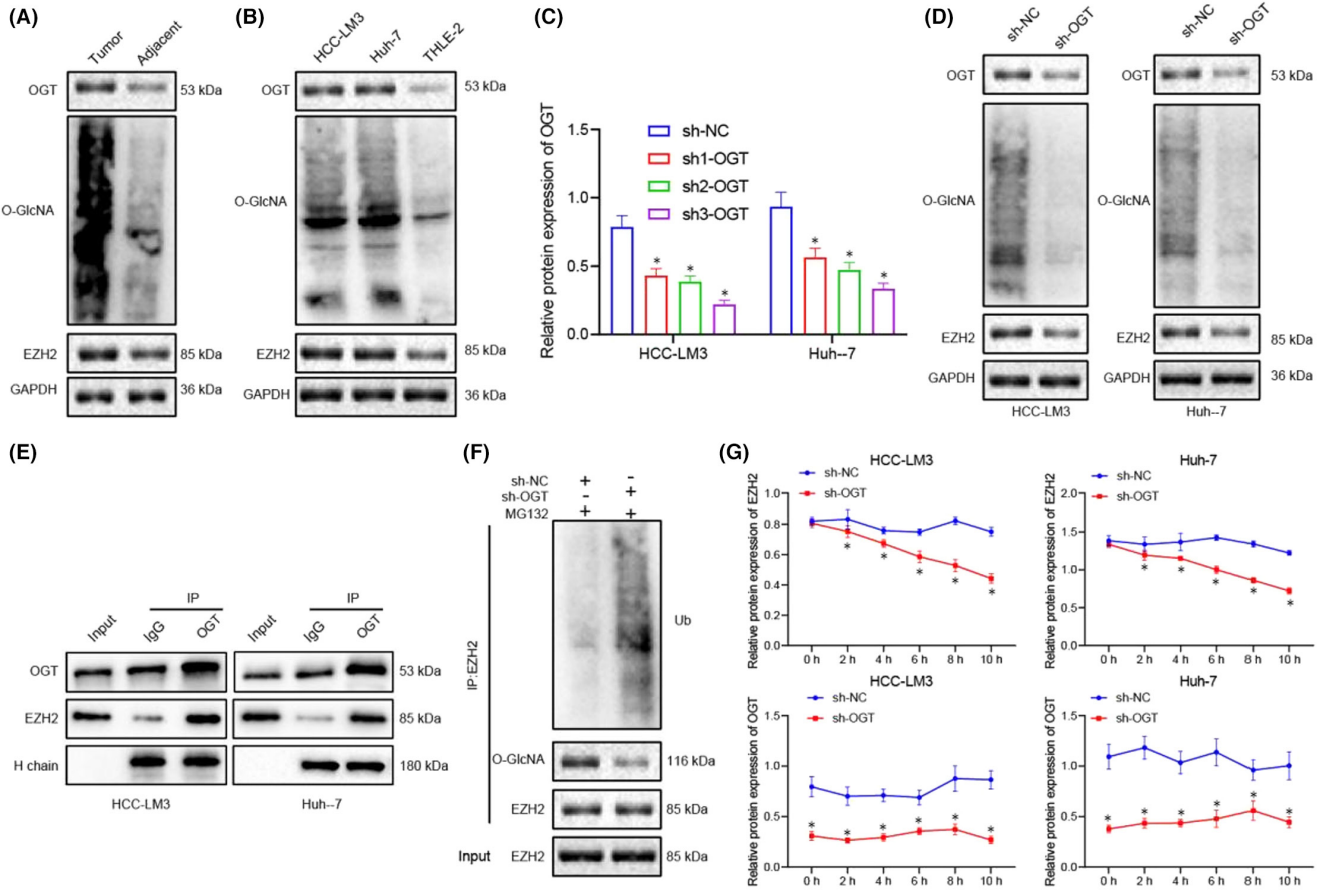
OGT‐mediated O‐GlcNAc stabilizes EZH2 and increases its expression. (A) Western blot for detecting the expression of OGT, O‐GlcNAc and EZH2 in HCC tissues and adjacent healthy tissues. (B) Western blot for analysing the expression of OGT, O‐GlcNAc and EZH2 in HCC cell lines, and HCC‐LM3 and Huh‐7 and normal liver cell line, THLE‐2. (C) Western blot and RT‐qPCR for detecting the expression of OGT in HCC‐LM3 and Huh‐7 cells of sh‐NC, sh1‐OGT, sh2‐OGT and sh3‐OGT. (D) The expression of OGT, O‐GlcNAc and EZH2 in HCC‐LM3 and Huh‐7 cells after silencing OGT. (E) The total lysate extracted from HCC cells was immunoprecipitated with OGT Ab, followed by Western blot analysis with designated antibodies with Ig heavy chain (H chain) as loading control. (F) Western blot analysis of ubiquitin and O‐GlcNAc on EZH2 immunoprecipitated with HCC cells stably expressing sh‐NC or sh‐OGT in the presence or MG132 (10 μM) for proteasome inhibition. (G) Detection of EZH2 and OGT protein levels treated with CHX (50 μg/ml) at different time points. Quantitative data were presented as mean ± SD. Data of two groups were processed using unpaired t test, and data among multiple groups were analysed via one‐way ANOVA and Tukey's post hoc test. *p < 0.05 compared with sh‐NC. Experiments were repeated 3 times

**FIGURE 6:**
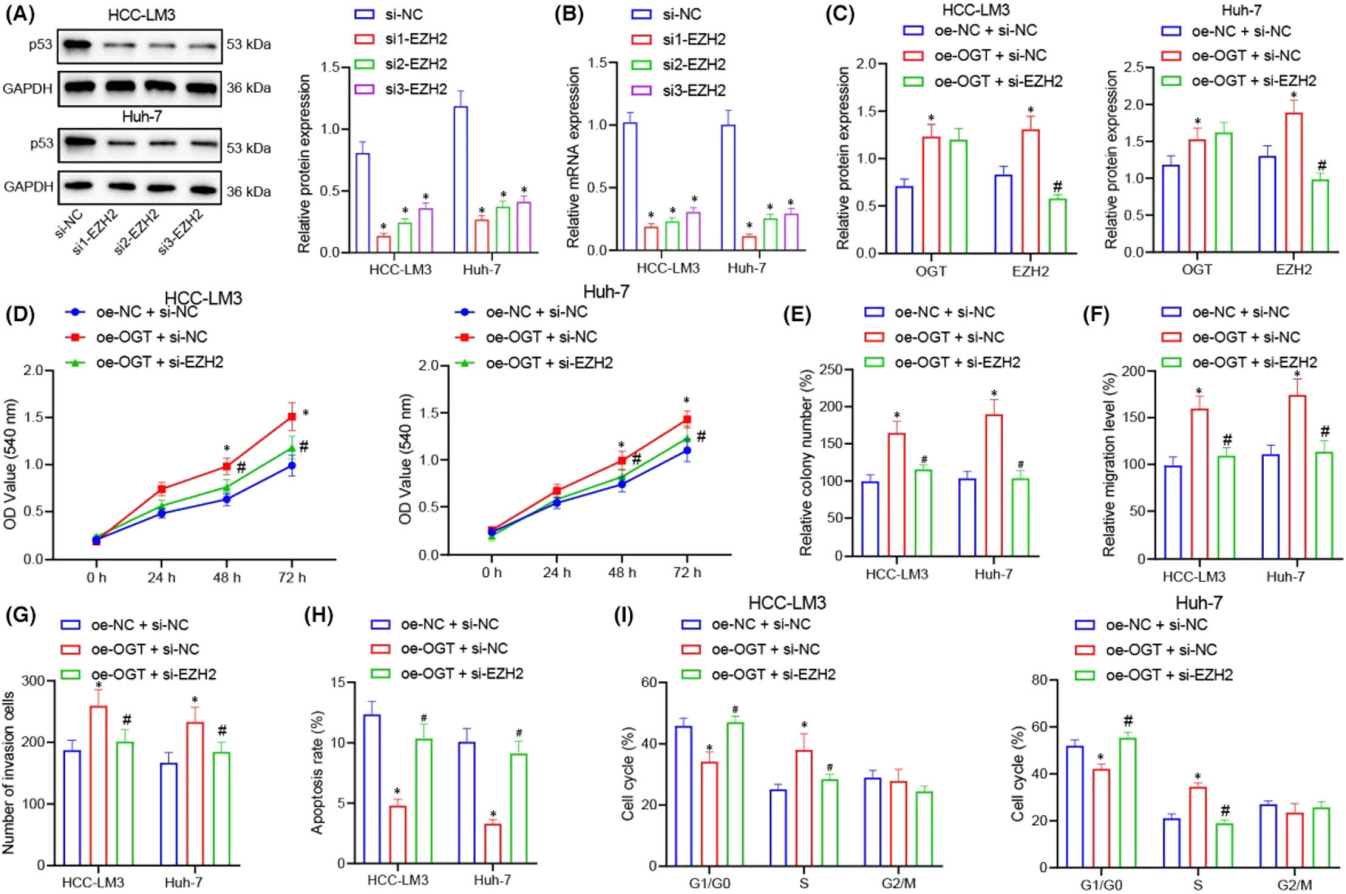
OGT promotes the proliferation, migration and invasion of HCC cells by regulating EZH2. (A and B) Western blot and RT‐qPCR for detecting the expression of EZH2 protein and mRNA in HCC‐LM3 and Huh‐7 cells of si‐NC, si1‐EZH2, si2‐EZH2 and si3‐EZH2 groups. (C) Western blot for detecting the expression of EZH2 protein and mRNA in HCC‐LM3 and Huh‐7 cells of response to oe‐NC +si‐NC, oe‐OGT+si‐NC and oe‐OGT +si‐EZH2. (D) MTT assay for detecting the proliferation of HCC‐LM3 and Huh‐7 cells in response to oe‐NC +si‐NC, oe‐OGT +si‐NC and oe‐OGT +si‐EZH2. (E) Colony‐forming unit assay for analysing the proliferation of HCC‐LM3 and Huh‐7 cells in response to oe‐NC +si‐NC, oe‐OGT +si‐NC and oe‐OGT +si‐EZH2. (F) Cell scratch assay for studying the migration of HCC‐LM3 and Huh‐7 cells in response to oe‐NC +si‐NC, oe‐OGT +si‐NC and oe‐OGT +si‐EZH2. (G) Transwell assay for studying the invasion of HCC‐LM3 and Huh‐7 cells in response to oe‐NC +si‐NC, oe‐OGT +si‐NC and oe‐OGT +si‐EZH2. (H) Flow cytometry for evaluating the cell apoptosis of HCC‐LM3 and Huh‐7 Cells in response to oe‐NC +si‐NC, oe‐OGT +si‐NC and oe‐OGT +si‐EZH2. (I) Flow cytometry for analysing the cell cycle of HCC‐LM3 and Huh‐7 cells in response to oe‐NC +si‐NC, oe‐OGT +si‐NC and oe‐OGT +si‐EZH2. Quantitative data were presented as mean ± SD. Data among multiple groups were analysed via one‐way ANOVA and Tukey's post hoc test. *p < 0.05 compared with si‐NC or oe‐NC +si‐NC, #*p* < 0.05 compared with oe‐OGT +si‐NC. Experiments were repeated 3 times

**FIGURE 7:**
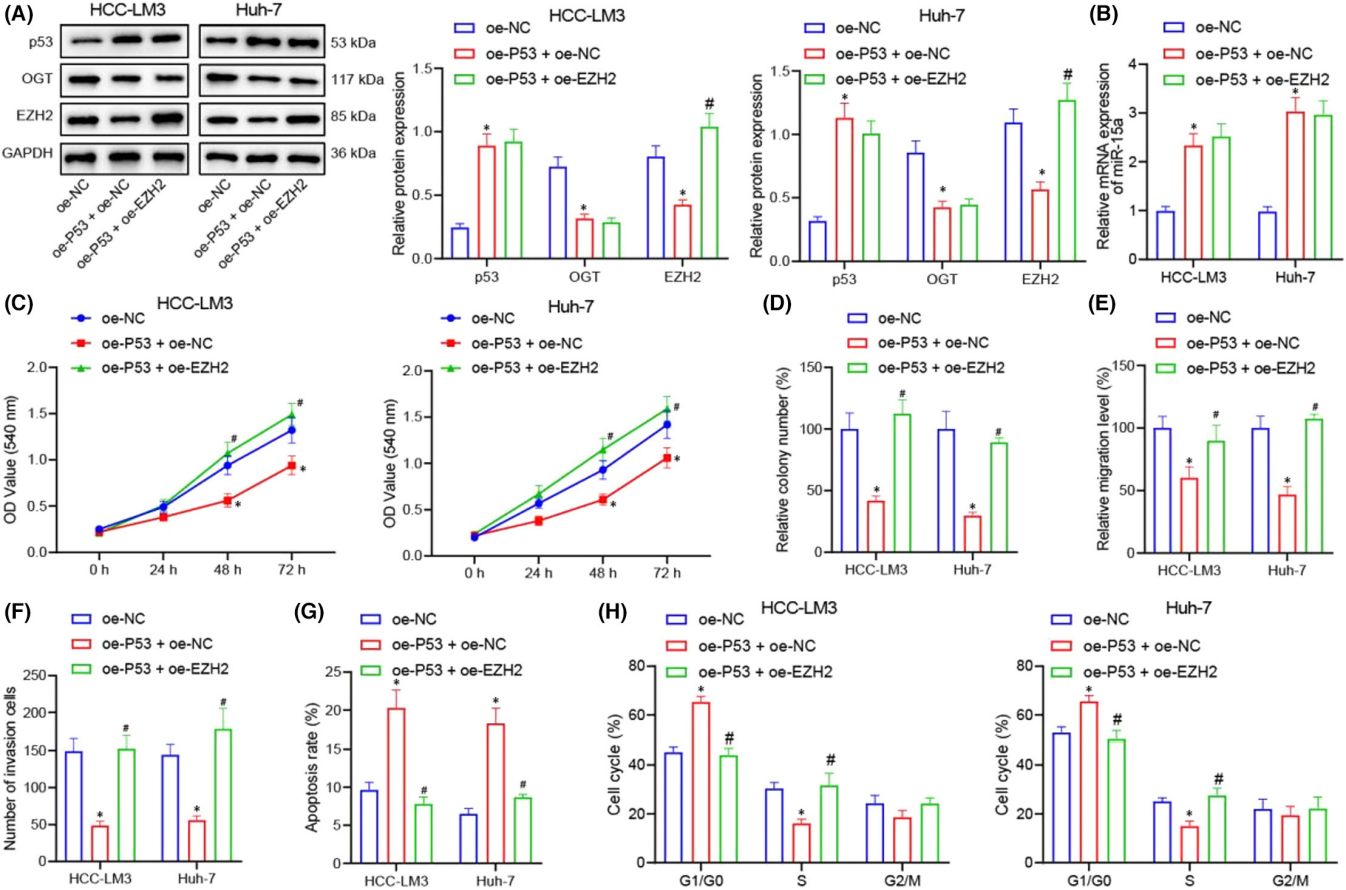
P53 inhibits the proliferation, migration and invasion and increases the apoptosis of HCC cells through miR‐15a/ OGT/EZH2 axis. (A) Western blot for detecting the expression of P53, OGT and EZH2 proteins in HCC‐LM3 and Huh‐7 cells in response to oe‐NC, oe‐P53 + oe‐NC and oe‐P53 + oe‐EZH2. (B) RT‐qPCR for detecting the expression of miR‐15a in HCC‐LM3 and Huh‐7 cells in response to oe‐NC, oe‐P53 + oe‐NC and oe‐P53 + oe‐EZH2. (C) MTT assay for detecting the proliferation in HCC‐LM3 and Huh‐7 cells in response to oe‐NC, oe‐P53 + oe‐NC and oe‐P53 + oe‐EZH2. (D) Colony‐forming unit assay for detecting the proliferation in HCC‐LM3 and Huh‐7 cells in response to oe‐NC, oe‐P53 + oe‐NC and oe‐P53 + oe‐EZH2. (E) Cell scratch assay for studying the migration in HCC‐LM3 and Huh‐7 cells in response to oe‐NC, oe‐P53 + oe‐NC and oe‐P53 + oe‐EZH2. (F) Transwell assay for studying the invasion in HCC‐LM3 and Huh‐7 cell in response to oe‐NC, oe‐P53 + oe‐NC and oe‐P53 + oe‐EZH2. (G) Flow cytometry for evaluating the cell apoptosis in HCC‐LM3 and Huh‐7 cells in response to oe‐NC, oe‐P53 + oe‐NC and oe‐P53 + oe‐EZH2. (H) Flow cytometry for analysing the cell cycle in HCC‐LM3 and Huh‐7 in response to oe‐NC, oe‐P53 + oe‐NC and oe‐P53 + oe‐EZH2. Quantitative data were presented as mean ± SD. Data among multiple groups were analysed via one‐way ANOVA and Tukey's post hoc test. *p < 0.05 compared with oe‐NC, #*p* < 0.05 compared with oe‐P53 + oe‐NC, and ns indicates no significant difference. Experiments were repeated 3 times

**FIGURE 8:**
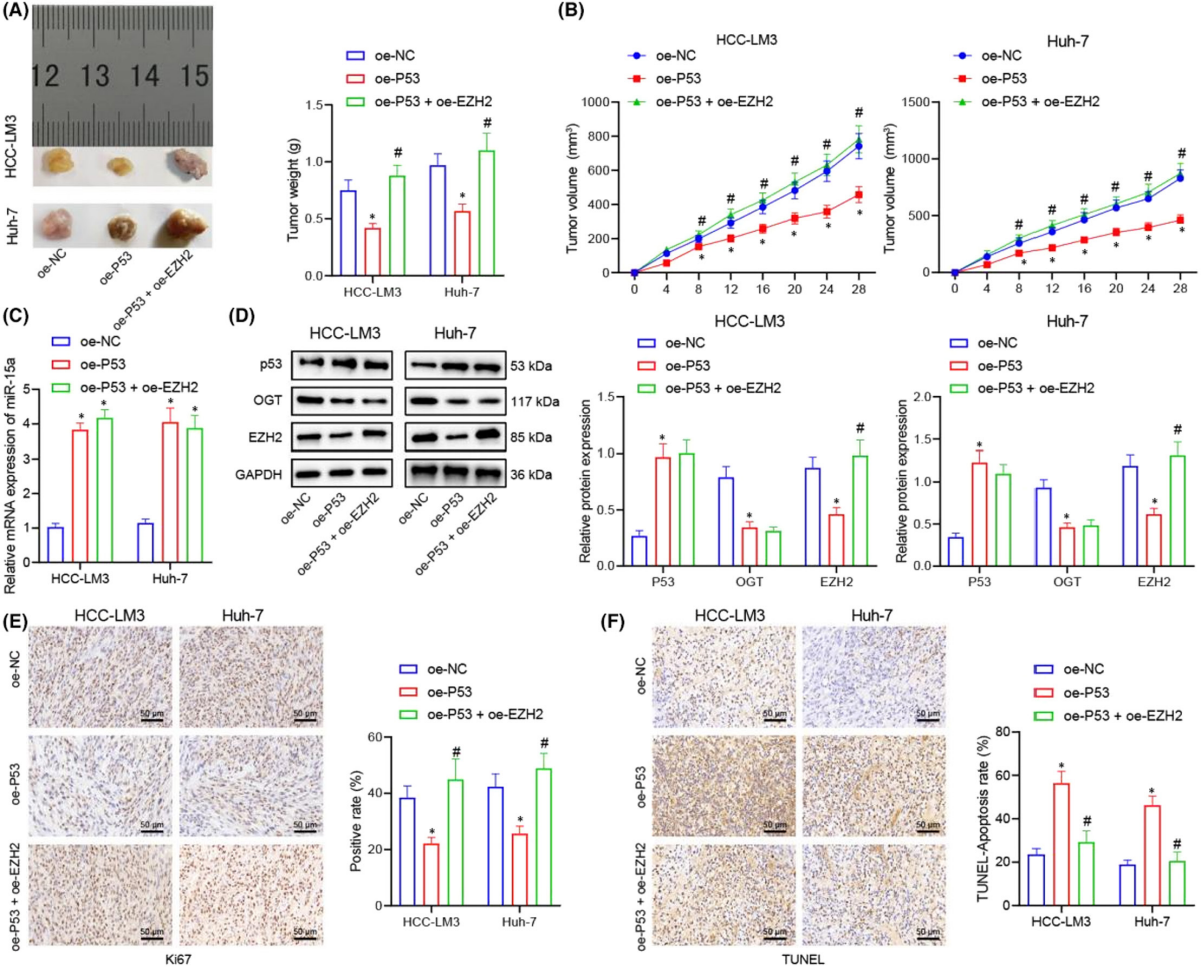
P53 suppresses the growth of xenograft liver tumour in nude mice through regulating miR‐15a/ OGT/EZH2 axis. (A) Tumour weights after 4‐week inoculation of HCC cells in nude mice, *n* = 6. (B) The trend of tumour sizes formed by HCC cells within 4 weeks in oe‐P53 + oe‐NC and oe‐P53 + oe‐EZH2 groups, *n* = 6. C, RT‐qPCR for detecting the expression of miR‐15a in tumours of nude mice in response to oe‐NC, oe‐P53 + oe‐NC and oe‐P53 + oe‐EZH2, *n* = 6. (D) Western blot for detecting the expression of P53, OGT and EZH2 in tumours of nude mice in response to oe‐NC, oe‐P53 + oe‐NC and oe‐P53 + oe‐EZH2. (E) Ki67 for analysing the proliferation of tumour cells. (F) TUNEL for analysing the apoptosis of tumour cells. *p < 0.05 compared with oe‐NC, #*p* < 0.05 compared with oe‐P53 + oe‐NC. Experiments were repeated 3 times

**FIGURE S2** Representative images of colony‐forming unit, scratch, Transwell assays and flow cytometry in the oe‐NC and oe‐P53 groups and si‐NC and si‐P53 groups. A. Representative image of colony‐forming unit assay for studying the proliferation of HCC‐LM3 and Huh‐7 cells in response to oe‐NC and oe‐P53 groups and si‐NC and si‐P53. B. Representative image of cell scratch assay for studying the migration of HCC‐LM3 and Huh‐7 cells of miR‐NC and miR‐15a‐mimics groups. C. Representative image of Transwell assay for studying the invasion of HCC‐LM3 and Huh‐7 cells in response to oe‐NC and oe‐P53 groups and si‐NC and si‐P53. D. Representative image of flow cytometry for evaluating the cell apoptosis of HCC‐LM3 and Huh‐7 cells in response to oe‐NC and oe‐P53 groups and si‐NC and si‐P53. E. Representative image of flow cytometry for analysing the cell cycle of HCC‐LM3 and Huh‐7 cells in response to oe‐NC and oe‐P53 groups and si‐NC and si‐P53. Quantitative data were presented as mean ± SD. Data of two groups were processed using unpaired t test, and data among multiple groups were analysed via one‐way ANOVA and Tukey's post hoc test. Data at different time points were analysed by repeated measures ANOVA and the Bonferroni post hoc test. * indicates *p* < 0.05 compared with oe‐NC and si‐NC, and ns represents no significant difference. Experiments were repeated 3 times
