# Supplementary figures and images for: P53 suppresses the progression of hepatocellular carcinoma via miR‐15a by decreasing OGT expression and EZH2 stabilization
Source: J Cell Mol Med. 2021 Sep 12;25(19):9168–82. doi: 10.1111/jcmm.16792 (PMC8500955; doi:10.1111/jcmm.16792)

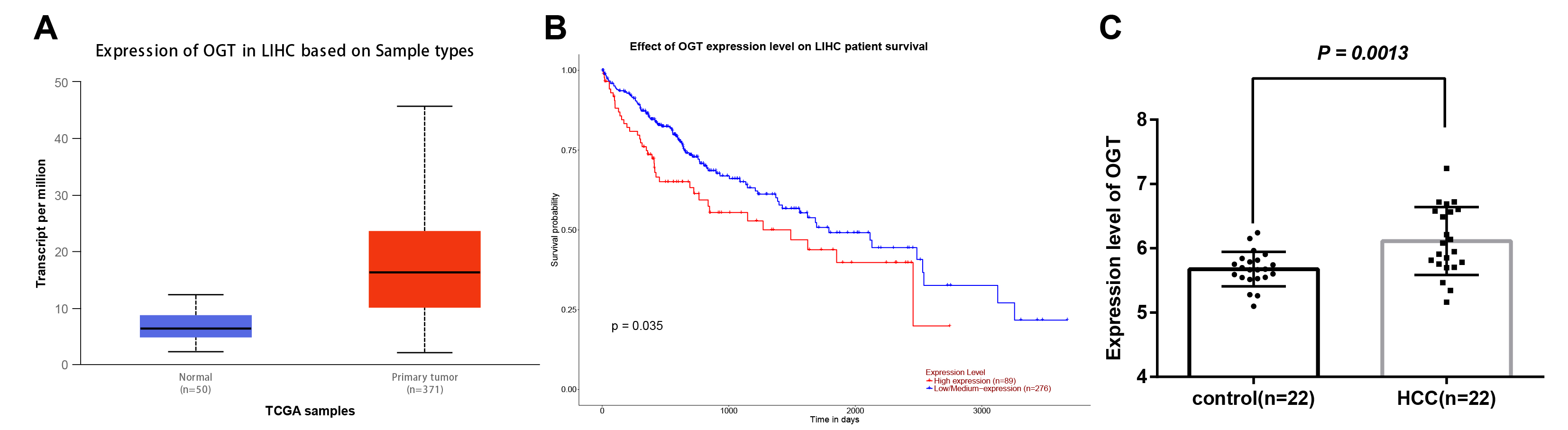

Supplement: Supplementary file 3 — Fig S3 [file JCMM-25-9168-s004.jpg]
