# Supplementary material for: P53 suppresses the progression of hepatocellular carcinoma via miR‐15a by decreasing OGT expression and EZH2 stabilization
Source: J Cell Mol Med. 2021 Sep 12;25(19):9168–82. doi: 10.1111/jcmm.16792 (PMC8500955; doi:10.1111/jcmm.16792)
Supplement: Supplementary file 5 — Table S1 [file JCMM-25-9168-s003.docx]

**TABLE S1. Primer list**

| Genes | Forward primer (5'-3') | Reverse primer (5'-3') |
| --- | --- | --- |
| OGT | TCCTGATTTGTACTGTGTTCGC | AAGCTACTGCAAAGTTCGGTT |
| EZH2 | TGGACCACAGTGTTACCAGCA | TGGGCGTTTAGGTGGTGTCT |
| miR-15a | TAGCAGCACATAATGGTTTGTG | GCGAGCACAGAATTAATACGAC |
| U6 | CTCGCTTCGGCAGCACA | ACGCTTCACGAATTTGCGT |
| GAPDH | TGGTGGGTATGGGTCAGAAGGACTC | CATGGCTGGGGTGTTGAAGGTCTCA |
